# From metabolic dysregulation to neurodegenerative pathology: the role of hyperglycemia, oxidative stress, and blood-brain barrier breakdown in T2D-driven Alzheimer’s disease

**DOI:** 10.1007/s11011-025-01700-z

**Published:** 2025-09-26

**Authors:** Ahmad Raza, Shafaq Saleem, Samar Imran, Sarah Rahman, Muhammad Haroon, Azeen Razzaq, Ahmad Hussain, Javed Iqbal, Brijesh Sathian

**Affiliations:** 1 Department of Medicine, Rawalpindi Medical University, Rawalpindi, Pakistan; 2 Department of Neurology, Fauji Foundation Hospital, Rawalpindi, Pakistan; 3 Punjab Medical College, Faisalabad, Pakistan; 4 Hamad Medical Corporation, P.O Box 3050, Doha, Qatar

**Keywords:** Type 2 diabetes mellitus (T2D), Alzheimer’s disease (AD), Insulin resistance, Amyloid-beta (Aβ) accumulation, Advanced glycation end products (AGEs), Neuroinflammation, Mitochondrial dysfunction

## Abstract

Type 2 Diabetes (T2D) and Alzheimer’s Disease (AD) share common risk factors that can be seen through T2D nearly doubling an individual’s likelihood of developing AD. Some AD patients show signs of metabolic dysfunction as well. This review focuses on the potential mechanisms associated with these two diseases, like insulin resistance, inflammation, oxidative damage, mitochondrial injury, and cell death. One of the notable elements in this connection is the “brain insulin resistance,” most frequently named as “type 3 diabetes,” which impairs glucose metabolism and facilitates amyloid beta (Aβ) plaque synthesis while reducing the action of insulin-degrading enzyme (IDE). Moreover, the overactivity of glycogen synthase kinase-3 beta (GSK-3β) also triggers taurine protein pathology. Raised concentrations of glucose in blood can produce advanced glycation end products (AGEs), which further exacerbate neuroinflammation in tandem with the mitigation of neurotoxic Aβ oligomers. Inflammation and subsequent damage to mitochondria lead to the dissolution of synapses. Current vascular insults include the breakdown of the blood-brain barrier (BBB) and decreased brain perfusion, along with other contributory factors to conditions conducive to neurotoxicity. Recently, novel therapies are emerging, including GLP-1 agonists, intranasal insulin, and mitochondrial antioxidants, that show surprising results for treating both conditions, but on the contrary, bioavailability and the timing of interventions remain a big challenge in the management of these diseases. Eventually, further research should center on understanding the mechanisms of integration along with the development of molecular biology, neuroimaging, and outcome-driven treatment strategies. Comprehensive strategies that exist between T2D-AD for integration and preservation of brain and metabolic health are addressed in this review.

**Graphical abstract:**

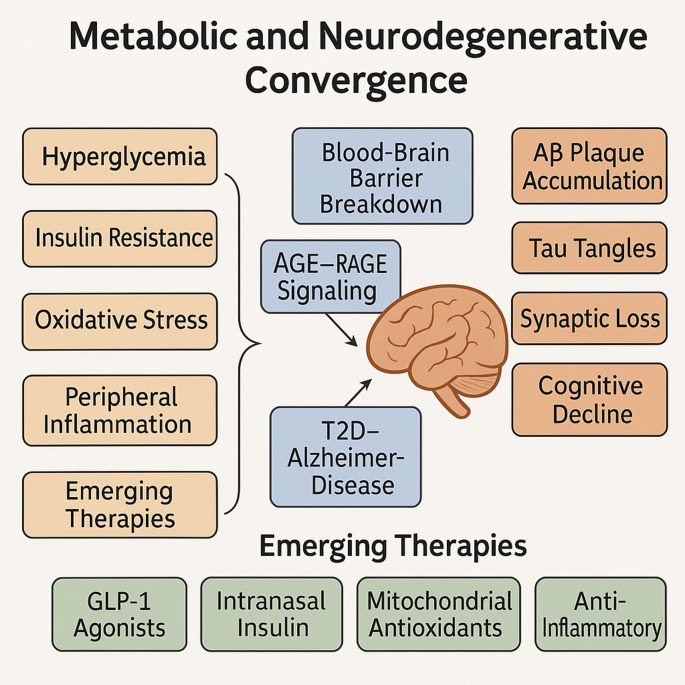

## Introduction

Diabetes mellitus and Alzheimer’s disease are two of the most prevalent chronic conditions in today’s world. Diabetes mellitus, especially type 2 diabetes (T2D), is identified by state of chronic hyperglycemia, which affected over 537 million people worldwide in 2021, and these numbers will increase to 643 million by 2030 due to a more relaxed approach to healthcare accessibility, aging, demographic shifts, and shifting lifestyle patterns (Hamzé et al. 2022). On the other hand. Alzheimer’s disease (AD), the most prevalent cause of dementia all over the world, is a progressive neurodegenerative disease that particularly affects the hippocampus. As of 2020, greater than 55 million individuals worldwide are suffering from dementia, a figure projected to nearly double to approximately 139 to 153 million by 2050 (Lipaj 2024). Due to the high prevalence of both of these diseases, it is crucial to understand the link between these two conditions. Studies have found correlations between T2D and AD. A 2024 meta-analysis by Cao et al., which pooled data from over 1.2 million participants, reported a 73% higher risk of dementia among individuals with T2D compared to non-diabetic controls (Cao et al. 2024). Additionally, patients diagnosed with AD are likely to suffer from changes in glucose metabolism.

Evidence is demonstrating that besides the more general common risk factors, such, such as age, hypertension, and dyslipidemia, there is a growing body of proof pointing towards pathological mechanisms that attempt to connect metabolic disorders with neurodegeneration. Both T2D and AD share common pathophysiologic mechanisms such as insulin resistance, neuroinflammation, and oxidative stress. Insulin resistance in T2D interferes with glucose metabolism and causes neuroinflammation, which leads to tau phosphorylation and amyloid-beta (Aβ) deposition, the two key features of AD. Furthermore, in T2D, the insulin-degrading enzyme (IDE), which breaks down insulin and Aβ, is less effective, further increasing the risk of cognitive deterioration (Mousavi et al. 2024).

**Fig. 1:**
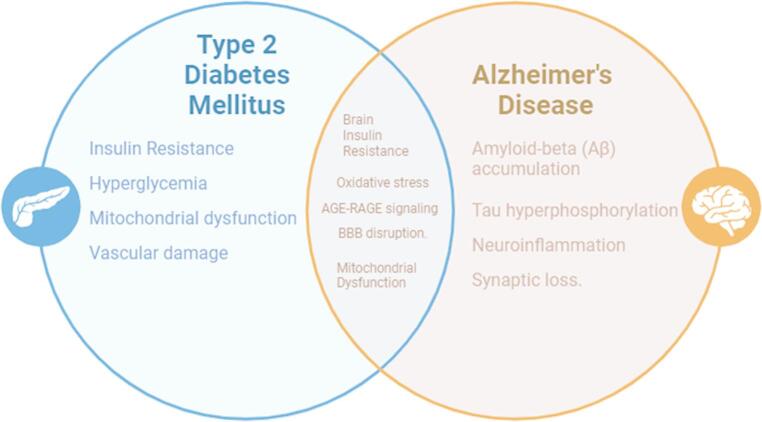
Shared pathophysiological pathways between type 2 diabetes and Alzheimer’s disease. A conceptual overview of overlapping mechanisms, including insulin resistance, oxidative stress, and neuroinflammation

A conceptual overview of the overlapping mechanisms is depicted in Fig. 1, given below.

Recent studies have also brought attention to the onset of late-onset Alzheimer’s Disease (LOAD) and the role that brain shrinkage and diabetic encephalopathy play in it. It is thought that AD in T2DM patients is caused by a combination of variables, including inflammasome activation, neuroimmunology, and abnormalities in brain perfusion; however, the precise processes are not entirely known (Lemche et al. 2025). Reactive oxygen species (ROS) and NADPH oxidase are also indicated to be important contributors to neuroinflammation and neuronal degeneration, indicating possible therapeutic targets for reducing cognitive loss in individuals with both AD and T2DM (Dash et al. 2025). Understanding these mechanisms of integration came with the development of molecular biology, neuroimaging, and the advancement of targeted therapeutic techniques aimed specifically at both these diseases. T2D and the interwoven pathways alongside the possible measures to mitigate the effects of this disease on cognition, are highlighted in this review, as briefly summarized in Table 1, given below.

**Table 1:** Advanced glycation end products (AGEs) in alzheimer’s disease: comparative levels and clinical correlations

Mechanism	Role in T2D	Role in AD	Key References
AGE-RAGE Signaling	Chronic hyperglycemia → AGE formation → oxidative stress	AGE-RAGE interaction stabilizes Aβ, amplifies neuroinflammation	Li et al. 2025
Mitochondrial Dysfunction	Reduced ATP production, pancreatic β-cell stress	ROS overproduction, impaired mitophagy, and synaptic loss	Wang et al. 2025
Insulin Resistance	Impaired glucose uptake, hyperinsulinemia	Brain glucose hypometabolism, Aβ accumulation, tau pathology	Khan et al. 2023
Vascular Dysfunction	Endothelial dysfunction, atherosclerosis, and BBB disruption	Hypoperfusion, reduced Aβ clearance, and BACE1 upregulation	Sweeney et al. 2018
Neuroinflammation	Systemic inflammation, TNF-α, IL-6 activation	Microglial activation, chronic neuroinflammation, Aβ/tau toxicity	Heneka et al. 2025

## Insulin resistance: a central player in T2D and AD

Beyond its quintessential role of blood glucose regulation, insulin plays many important functions within the brain. Insulin supports neurogenesis, synaptic plasticity, and signaling pathways activation, including PI3K/Akt/mTOR, along with neuronal survival, especially in the key areas that are important for cognition, such as the hippocampus, prefrontal cortex, and hypothalamus. Insulin receptors (IRS) present in these areas facilitate the release of neurotransmitters, like acetylcholine and glutamate, which play crucial roles in memory building and learning. Proper insulin signaling and functioning prevents the formation of senile plaques and also promotes the integrity of mitochondria, hence providing neuroprotection against the development of AD (Yaribeygi et al. 2023)..

Alzheimer’s disease (AD) is known to be a multi-factorial neurodegenerative disorder with some element of genetic and environmental factors involved in its development (Saragea 2024). Based on the current paper focusing on mechanisms associated with late-onset Alzheimer’s disease (LOAD)—i.e., mitochondrial dysfunction, insulin resistance, oxidative injury—these factors were not known to only characterize LOAD. Shared downstream pathological events are observed in familial early-onset AD (EOAD)—representing a smaller proportion of cases and commonly involving mutations in the *APP*, *PSEN1*, or *PSEN2* genes—amassing amyloid, tau pathology, and synaptic loss (Oliver & Reddy, 2019). Unlike EOAD, where the genetic alterations are unique mutations due to transgenic genes, LOAD is more strongly associated with risk alleles such as the *APOE ε4* and a chronic combination of metabolic states promoting a general system and cerebral bioenergy collapse (Saragea 2024). Thus, while we review sporadic LOAD in the current work, the molecular processes described may also overlap with EOAD at disease progression and neuronal compromise. The basic bridge between metabolic dysfunction and neurodegeneration is dysregulation of insulin signaling, specifically through the PI3K/AKT/mTOR pathway, hence causing both metabolic and neurodegenerative disorders (Liu et al. 2025). This understanding reflects a broader shift in how Alzheimer’s Disease is conceptualized — not merely as a neurodegenerative illness but as a manifestation of systemic metabolic dysfunction in the brain.

Increasing evidence indicates that disturbances in brain energy balance, mitochondrial integrity, oxidative regulation, and amino acid metabolism all converge to produce AD pathology, supporting the view that classical markers like amyloid plaques and tau tangles may be downstream consequences of these deeper metabolic failures (Polis and Samson 2019). The concept of Alzheimer’s Disease as “Type 3 Diabetes” has gained strong scientific support due to the overlap between brain-specific insulin resistance and hallmark AD pathology. Disrupted insulin and insulin-like growth factor (IGF) signaling within the brain impairs glucose metabolism, promotes amyloid-beta accumulation, and contributes to progressive neurodegeneration — features characteristic of both metabolic and cognitive decline (de la Monte and Wands 2008). Research on AD patient brains after death demonstrated that the density of IR receptors in the hippocampus decreases by 50–80% which shows how intimately neurodegeneration and insulin dysfunction are linked. Insulin resistance causes over-activation of BACE1 in the brain, which not only causes increased production of amyloid beta plaques but also lowers the activity of insulin-degrading enzyme (IDE) to remove Aβ effectively (Yoon et al. 2023). Insulin resistance also causes the disruption of hyperphosphorylation of tau signaling and leads to activation of Glycogen Synthase Kinase-3β (GSK-3β), which causes abnormal phosphorylation of tau protein, forming neurofibrillary tangles, a key pathological hallmark of AD (Hobday and Parmar 2021). Chronic neuroinflammation, which is mediated by proinflammatory cytokines such as TNF-α and IL-6, further complicates the neuronal insulin signaling, hence impairing glucose metabolism and further promoting neurodegeneration (Wang et al. 2022). Furthermore, studies have shown that oligomeric Aβ1–40 reduces the expression of insulin receptors (IR) on the neurons by directly inhibiting insulin receptor phosphorylation and further leads to the progression of both AD and insulin resistance (Molina-Fernández et al., 2022).

**Fig. 2:**
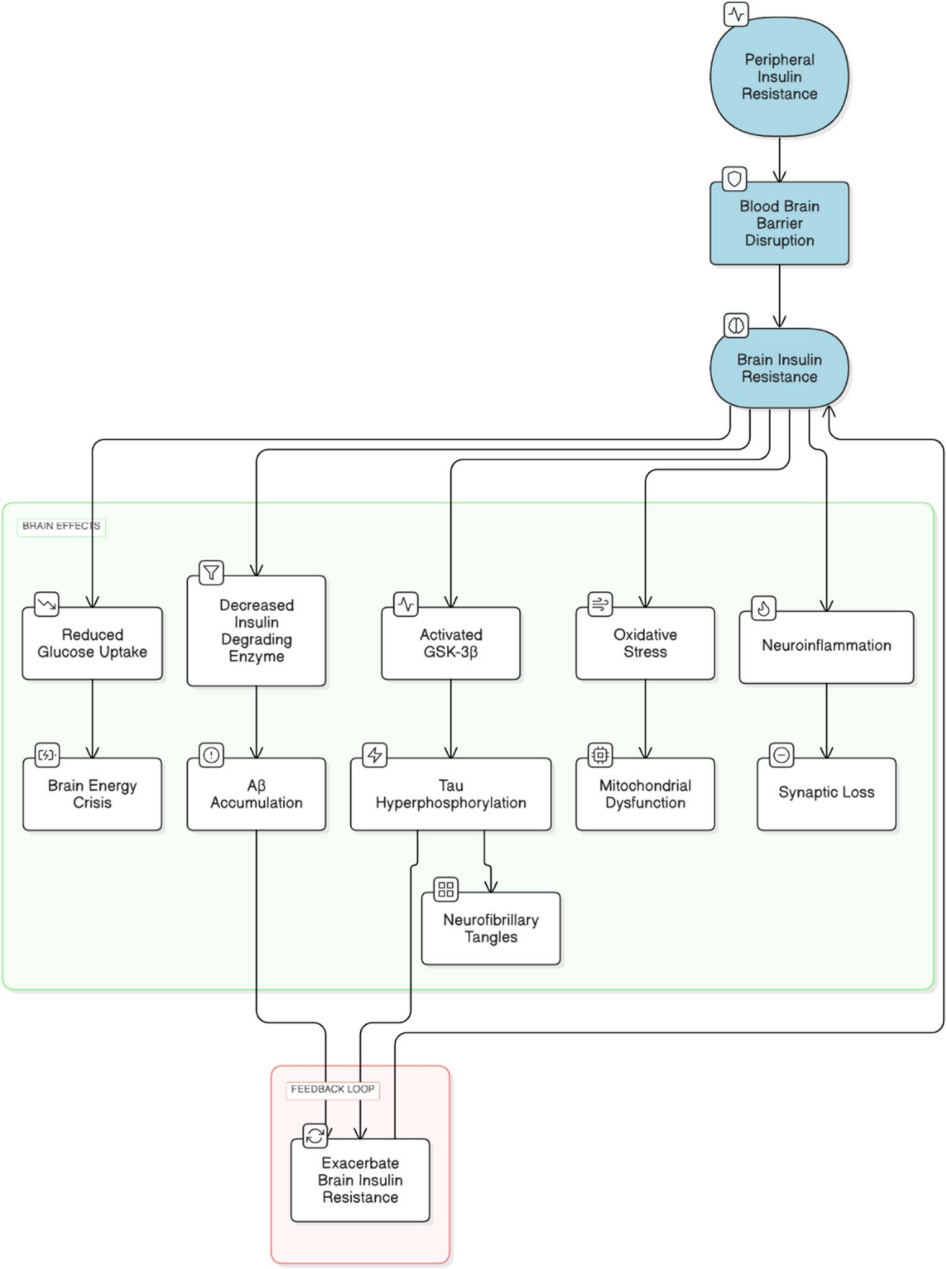
Brain insulin resistance and its role in Aβ Accumulation and Tau pathology. Schematic representation of impaired insulin signaling pathways contributing to neurodegeneration

Type 2 diabetes (T2D) also has effects on the brain via peripheral insulin resistance. In T2D, sustained hyperinsulinemia results in reduced brain ability to transport insulin through the blood-brain barrier (BBB), thereby compromising the brain’s insulin availability and sensitivity. This dysfunction leads to the complex cycle of metabolic dysregulation, oxidative stress, and neuroinflammation, thus contributing to cognitive decline and increasing AD risk in T2D patients (Abdalla 2024). Postmortem of AD patients also reveals that there is decreased insulin-stimulated phosphorylation of key insulin signaling molecules, which supports the role of defective insulin pathways in neurodegeneration (Reid et al. 2025). Hence, this connection underscores the importance of managing insulin resistance through antidiabetic drugs for not only managing the blood sugars but also for the prevention and slow progression of AD (Kciuk et al. 2024). A schematic representation is given below in Fig. 2.

## Hyperglycemia and advanced glycation end products (AGEs): A glycation-Inflammation axis

### Chronic hyperglycemia: A catalyst for neurotoxicity

Chronic elevated blood glucose levels, a hallmark of Type II diabetes (T2D), when left uncontrolled, may accelerate the neurodegenerative pathology through oxidative stress, aberrant mitochondrial function, increased inflammatory response, and BBB breach. Chronically high blood glucose levels lead to overproduction of ROS through polyol metabolism and increased activity of the mitochondria, causing damage to neurons, blood vessels, and blood antioxidant systems (Tomić 2020). This disruption further worsens the neurodegenerative processes, especially AD. The hyperglycemia disrupts the BBB, which increases its permeability, making it easier for damaging proteins, inflammatory factors, and blood-borne amyloid-beta peptides to infiltrate the brain (Wątroba et al. 2023). This increased Aβ accumulation not only hinders the clearance of Aβ but also facilitates its retention within the body, which leads to the escalation of AD (Moussiopoulou et al. 2025). Moreover, sustained hyperglycemia also decreases the efficiency of supportive cells such as astrocytes and microglia, which maintain inflammation for an extended period of time (Gao et al. 2024). As a result, this inflammation is detrimental to the cognitive deterioration associated with AD.

### AGE-RAGE signaling: A molecular Bridge between T2D and AD

Non-enzymatic attachment of glucose with lipids, proteins or nucleic acids forms Advanced glycation end products (AGEs), a process expedited in the state of hyperglycemia. Likewise, the receptor for advanced glycation end products (RAGE) is also upregulated in a state of hyperglycemia, disrupting the Aβ clearance, hence contributing to the development of AD (Cruz et al. 2023). The AGE and RAGE receptors bind and activate the key inflammatory pathways in neurons and glial cells, instigating oxidative stress and inflammatory changes in the brain via the activation of NF-κB and NADPH oxidase supporting pathways (Pinkas and Aschner 2016). This cascade further heightened the neuroinflammation by the overexpression of proinflammatory cytokines such as IL-1β, TNF-α, and COX-2, all of which are in an elevated state and promote neuronal damage (Springer 2022). AGE facilitates amyloid plaque formation in the brain by impairing the breakdown and also stabilizing the Aβ fibrils at the same time, thus contributing to Aβ accumulation and neurotoxicity (Koerich et al. 2022). Additionally, the AGE-RAGE interaction leads to tau hyperphosphorylation and neurofibrillary tangle formation through the GSK-3β activation, another hallmark of AD pathology (Cross et al. 2024). Hence, these AGE-RAGE signaling pathways play a vital role in the emergence and clinical progression of AD and also hint at the possibility that targeting these pathways would be a cornerstone to slow cognitive decline and progression in AD patients (Twarda-Clapa et al., 2022). The key findings are summarized in Table 2, given below.

**Table 2:** Advanced glycation end products (AGEs) in alzheimer’s disease (AD): elevated cerebrospinal fluid (CSF) AGE-RAGE interaction and serum AGE levels correlate with Aβ plaque severity and cognitive decline

AGE Measurement	AD vs. Control (AGE Levels)	Mechanistic Insight	Clinical Relevance	Reference
CSF AGE-RAGE interaction	3× higher in AD	Activates NF-κB → inflammation	Correlates with plaque burden	Chakraborty et al. 2021
Serum AGEs	2.5× higher in AD	↓ Insulin signaling, ↓ Aβ clearance	Predicts cognitive decline and memory impairment	Zhang et al. 2023
Brain AGE deposits	↑ staining in hippocampus & cortex	Stabilizes Aβ, ↑ tau phosphorylation	Indicates regional neurodegeneration	Koerich et al. 2022
Plasma AGEs	↑ in Mild Cognitive Impairment (MCI) & AD vs. controls	↑ RAGE, BBB dysfunction	Early biomarker for MCI→AD	Avitan et al. 2021

## Inflammation and oxidative stress: the fire and the spark

Metaflammation, more accurately identified as chronic inflammation, is a major contributor to both Alzheimer AD and T2D. In T2D, long-term metabolic stress promotes the secretion of inflammation mediators such as TNF-α, IL-6, and IL-1β from adipose and immune cells. These cytokines cross the BBB and activate microglia, which sustain further neuroinflammation. In AD, activated microglia are a major contributor to brain pathology. Instead of clearing amyloid-beta (Aβ) deposits, chronically active microglial cells take on a pro-inflammatory phenotype, exacerbating AD neurodegeneration by more intensely damaging neurons and so increasing AD progression. Increased neuroinflammation in AD patients with coexisting T2D suggests the linkage between peripheral and central immune dysfunction (Zhang et al., 2023). Systemic inflammation also reduces the BBB integrity, increasing the permeability of neurotoxic inflammatory factors, which intensifies neurodegeneration in AD (Sun et al. 2022). Cytokine signaling blockade (ex, IL-1β inhibitors) and targeted dampening of microglial activation by specific inhibitors, CSF1R blockers, have shown efficacy in controlling the inflammation and thus serving some therapeutic effects for AD and T2D patients (Zhou et al. 2024).

Chronic insulin resistance in the periphery, seen with T2D, stimulates chronic production of proinflammatory mediators TNF-α, IL-6, and IL-1β from adipose and immune cells. Increasing appreciation places adipose tissue as an immunometabolic organ; thus, its dysfunction in states of obesity and T2D most likely enhances NLRP3 inflammasome-mediated secretion of IL-1β to decrease insulin sensitivity (Söderbom and Zeng 2020). This promotes system-wide inflammation. These circulating cytokines cross a compromised blood-brain barrier (BBB) with microglial activation through NF-κB and NADPH oxidase pathways, thereby increasing oxidative stress, disrupting mitochondria as well, and causing neuronal damage. Aβ oligomers within CNS neurons dissociate mitochondrial complexes I and III to increase ROS generation that is perpetually neurotoxic. Of note here is the fact that hyperglycemia, ROS, and Aβ trigger the NLRP3 inflammasome to engage caspase-1-dependent synaptotoxic IL-1β release as well as pyroptosis, thus linking metabolic inflammation to neurodegeneration in AD and other neurodegenerative diseases (Wu et al. 2020).

A major player in the development of T2D and AD is oxidative stress, which is marked by unbalanced ROS and antioxidant defenses. In T2D, high blood sugar triggers oxidative stress through several mechanisms such as glucose autoxidation, Protein Kinase C (PKC) activation, and polyol pathway overactivity, which results in cellular damage and insulin resistance. Protein Kinase C (PKC) activation contributes to endothelial dysfunction, while Polyol Pathway overactivation depletes crucial antioxidants like glutathione, further worsening the oxidative stress (Karthikeyan et al. 2022). In case of AD, amyloid-beta (Aβ) oligomers are responsible for the generation of hydroxyl radicals, which cause mitochondrial degeneration and an endless cycle of oxidative damage. Higher amounts of damaged mitochondria lead to further ROS production, which hastens neuronal degeneration. Some of the most promising agents for reducing neuronal damage are those that mitigate oxidative stress with mitochondrial antioxidants like MitoQ and CoQ10. Moreover, targeting NRF2 may help in augmenting the already existing defensive mechanisms of antioxidants and increasing neuronal health, which is highly beneficial to T2D and AD patients (Ni and Wu 2021). Besides T2D and AD, oxidative stress additionally has been implicated in a plethora of degenerative comorbidities, which are extremely prevalent in Alzheimer’s disease, including cardiovascular disorders, metabolic syndrome, osteoporosis, arthritis, and certain neuropsychiatric diseases.

The overlapping of these disease pathways suggests that reactive oxygen species (ROS) act as a unifying pathological mechanism across multiple age-related disorders. The ubiquitous participation of ROS in promoting cell damage highlights the therapeutic value of antioxidants not only for cognitive preservation in AD but also in the improvement of the prognosis of its associated comorbidities (Avitan et al. 2021).Besides T2D and AD, oxidative stress additionally has been implicated in a plethora of degenerative comorbidities, which are extremely prevalent in Alzheimer’s disease, including cardiovascular disorders, metabolic syndrome, osteoporosis, arthritis, and certain neuropsychiatric diseases. The overlapping of these disease pathways suggest that reactive oxygen species (ROS) act as a unifying pathological mechanism across multiple age-related disorders. The ubiquitous participation of ROS in promoting cell damage highlights the therapeutic value of antioxidants not only for cognitive preservation in AD but also in the improvement of the prognosis of its associated comorbidities (Avitan et al. 2021).

## Mitochondrial dysfunction: energy crisis in neurons

### Impaired mitochondrial biogenesis and energy deficits

Mitochondria, the powerhouse of the cell, generate the energy needed for the proper functioning of the body. In T2D, insulin resistance leads to the inhibition of peroxisome proliferator-activated receptor gamma coactivator 1-alpha (PGC-1α), which is needed for biogenesis of mitochondria and ATP synthesis. Multiple studies on AD patient brains show that the lower expression of PGC-1α is linked to cognitive decline in patients with AD (Paul, 2021). The cellular energy production is reduced due to impairment in mitochondrial biogenesis and results in the depletion of neuronal energy along with increased oxidative stress, so it acts as a critical link between T2D and AD development.

A complication often described in both T2D and AD is having lower proficiency in oxidative phosphorylation (OXPHOS), which is a factor for the energy shortage and the degeneration of mitochondria. Defects were found in the electron transport chain (ETC) complexes, especially Complexes I, III, and IV, during postmortem of AD patients, and are identical to existing mitochondrial problems studied in T2D. This reduction in the OXPHOS further causes the energy deficiency and accelerates the neurodegeneration process in both conditions (Iheagwam et al. 2025; Shaaban et al.,^2025^). Hence, this complex interaction between the metabolic disorder and neurodegenerative ailment shows that mitochondrial capacity reduction is the central piece in the development of both of these conditions (Koshatwar et al. 2023).

### Oxidative stress and mitochondrial damage

Mitochondrial dysfunction and oxidative stress are the central pillars in both T2D and AD development. As discussed earlier, oxidative stress driven by chronic hyperglycemia disrupts mitochondrial redox balance and antioxidant defenses, further amplifying cellular injury in T2D and AD (Potenza et al. 2021). These perturbations in mitochondrial activity aid in the worsening insulin dependency, induce reactive insulin surge, and lead to multi-system dysfunctions, exerting further entrenchment of the disease.

In AD, amyloid-beta has also been shown to impair mitochondrial integrity, contributing to downstream apoptotic signaling, a process explored in more detail earlier. In addition, strongly phosphorylated tau blocks mitochondrial movement, disrupts synaptic recovery, and energy release. Deficient mitophagy resulting from inactive PINK1/Parkin signaling causes the build-up of dysfunctional mitochondria, aggravating neurodegenerative processes (Chakravorty et al. 2019). Mitochondrial and oxidative stress cycles impairing AD neurodegeneration are viciously interlocked. The pathological links of T2D and AD accentuate the interplay of diseases with profound impact of advanced age and dependency on mitochondrial activity, as shown in Table 3, given below.

**Table 3:** Mitochondrial dysfunction in type 2 diabetes and alzheimer’s disease: contrasting mechanisms in pancreatic β-Cells and neurons

Parameter	T2D (Pancreas)	AD (Neurons)
ROS Production	↑ via glucose autoxidation, ETC dysfunction	↑ via Aβ-mitochondria interaction, oxidative stress
Biogenesis	↓ PGC-1α expression → reduced mitochondrial formation	↓ PGC-1α expression → impaired energy metabolism
Mitophagy	Impaired due to oxidative stress and chronic hyperglycemia	Defective PINK1/Parkin signaling → accumulation of damaged mitochondria

### Calcium dysregulation and apoptosis

**Fig. 3:**
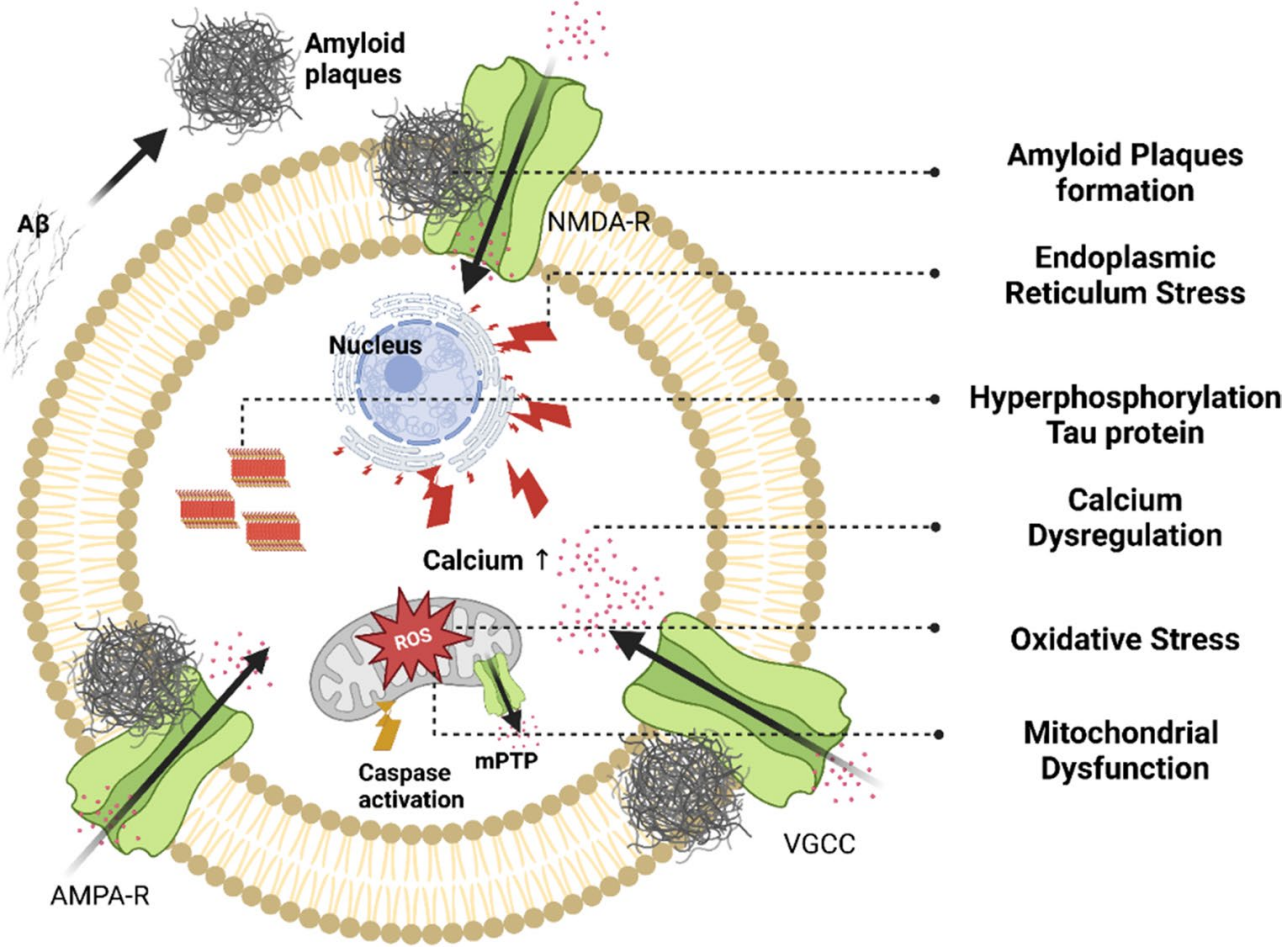
Mitochondrial calcium overload and apoptotic pathways in T2D and AD. Mechanisms linking calcium dysregulation to neuronal and pancreatic β-cell death

In the cell, mitochondria is the main calcium regulator and its dysregulation leads to the progression of T2D and AD. Insulin resistance in T2D causes calcium overload in mitochondria, which in turn opens the mitochondrial permeability transition pore (mPTP), consequently causing cell death (Piao et al. 2024). Figure 3 depicts this mechanism below.

While in the case of AD, excessive mitochondrial calcium uptake is the main culprit that causes disruption of metabolic activity, increasing ROS production, and cell death by apoptosis (Jadiya et al. 2024). And also, amyloid-beta (Aβ) interferes with calcium signaling and leads to mitochondrial calcium accumulation, strongly indicating neuronal death (Garcia-Casas et al. 2023). Such a combination of mechanisms classifies the phenomenon as calcium toxicity in AD’s progression.et al., 2023). Such a combination of mechanisms classifies the phenomenon as calcium toxicity AD’s progression.

Dysregulation of calcium amplifies cognitive impairment and neurodegeneration due to oxidative stress and synaptic plasticity reduction, as observed in AD patients (Godoy et al. 2021). Strongly phosphorylated tau inhibits mitochondrial transport, impairing energy metabolism and maintenance of synapses. Also, mitophagy due to malfunctioning PINK1/Parkin signaling causes the accumulation of damaged mitochondria and heightens vulnerability to neurodegeneration, as summarized in Table 4.

**Table 4:** Oxidative stress (ROS) and mitochondrial dysfunction in T2D and AD: ROS elevation via glucose autoxidation (T2D) and Aβ-mitochondria interactions (AD), with mitochondrial biogenesis decline via PGC-1α reduction in pancreatic β-cells (T2D) and neurons (AD)

Parameter	T2D(Pancreas)	AD (Neurons)
ROS Production	↑ Glucose Autoxidation	↑ AB-mitochondria interaction
Biogenesis	↓ PGC-1α → fewer mitochondria	↓ PGC-1α → impaired energy

Approaches to restore mitochondrial function, such as mitophagy activators, PGC-1α agonists, and calcium modulators, demonstrate potential for reestablishing calcium balance, improving mitochondrial bioenergetics, and protecting neuronal health (Walters and Usachev 2023).

## Amyloid-Beta and tau: converging pathways

Type 2 diabetes has a significant impact on Alzheimer’s disease pathology as it accelerates the amyloid-beta (Aβ) accumulation through several interconnected mechanisms. Insulin resistance, as highlighted in earlier sections, reduces amyloid-beta clearance due to IDE competition and enhances its production through BACE1 activation (Rowland et al. 2023). Additionally, high blood sugar and JNK signaling boost β-secretase (BACE1) expression, which ultimately increases the production of Aβ from its precursor protein (Ricke 2022). The advanced glycosylation end products (AGEs) are known to cause an inflammatory response by interacting with RAGE receptors and increasing the Aβ toxicity. Studies suggest that soluble Aβ oligomers are particularly harmful as they disrupt synaptic function before plaques even form, leading to early cognitive decline (Patel et al. 2022).

### Tau hyperphosphorylation

**Fig. 4:**
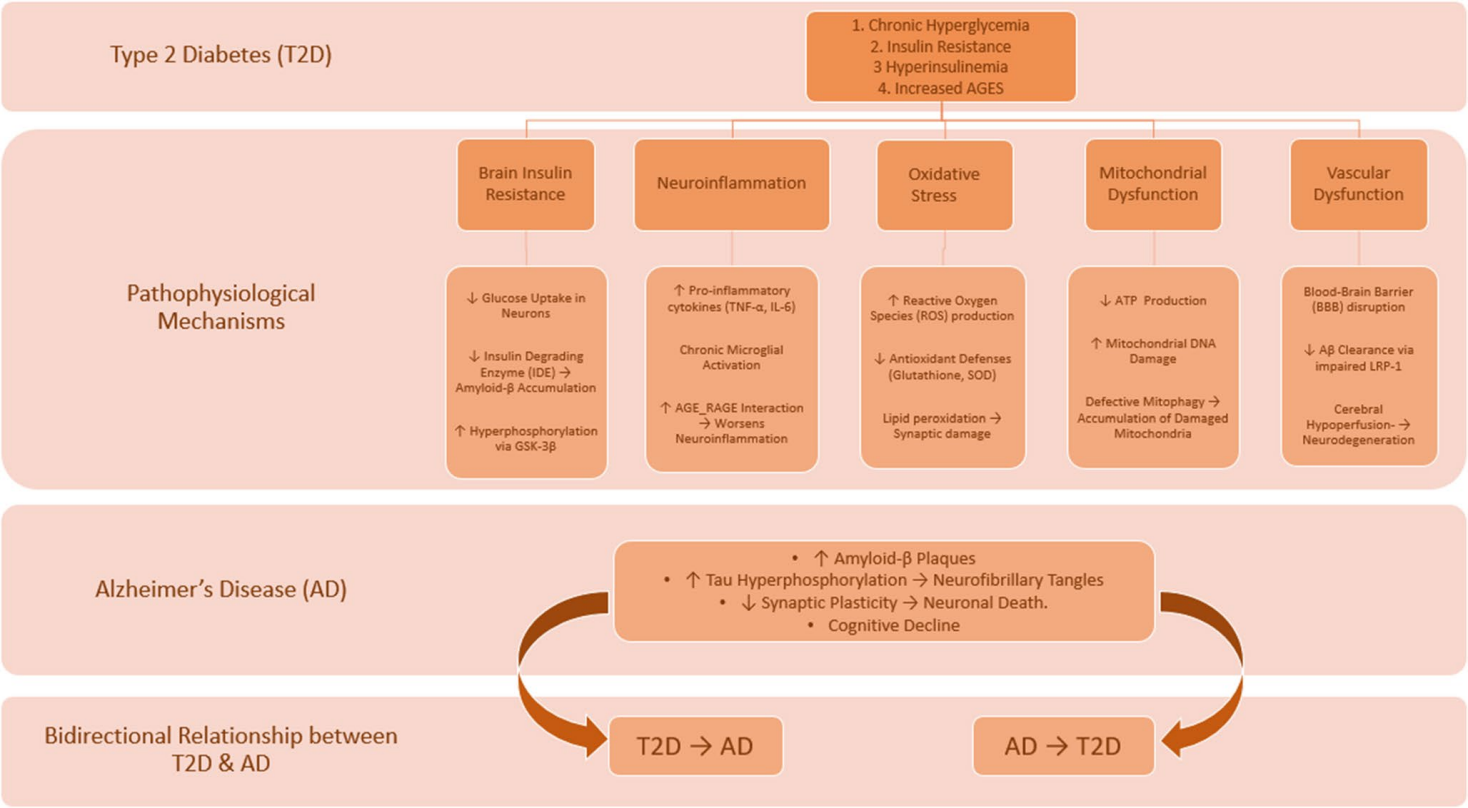
Pathogenic synergy: Hyperglycemia-driven amyloid-beta aggregation and Tau hyperphosphorylation in T2D-AD Comorbidity

While amyloid-beta accumulation initiates early neurotoxic events in Alzheimer’s disease, tau hyperphosphorylation plays a central role in driving neuronal dysfunction and disease progression. Amyloid-beta and tau are mechanistically linked, as Aβ aggregation accelerates tau misfolding, phosphorylation, and spread, amplifying neurotoxicity and cognitive decline (Busche and Hyman 2020). In type 2 diabetes, insulin resistance affects the regulation of tau proteins through different mechanisms. One of the most important pathways is upregulation of GSK-3β. This causes phosphorylation of tau protein at specific sites that leads to neurodegeneration (Vijayam et al. 2022). Also, the tau protein hyperphosphorylation results in aggregation or clumping of tau and is further exacerbated by inflammation and oxidative stress. In short, these pathways lead to destabilization of tau protein structure and function (Singh et al. 2024). The destabilized tau protein is unable to play its part in mitochondrial transport and leads to energy deficit and synaptic dysfunction. As a result, this abnormally phosphorylated tau accelerates neuronal degeneration (El Idrissi and Alonso, 2022). Studies done on cerebrospinal fluid of T2D patients reveal the presence of high levels of phosphorylated tau proteins and link them with faster cognitive decline (Ma et al. 2025), as also depicted in Fig. 4.

## Vascular dysfunction: the overlooked culprit

Type 2 diabetes has a major impact on cerebrovascular health. T2D causes atherosclerosis and microvascular damage along with cerebral hypoperfusion, and so decreases the brain’s energy supply. Atherosclerosis is the hardening of both small and large blood vessels, which decreases the blood flow. The decreased cerebral blood flow impairs the neurovascular coupling and so increases the risk of AD (Eyre et al. 2022). Secondly, T2D plays its role in microvascular damage and loss of capillary structure, which in turn reduces the oxygen supply and causes hypoxia. This further deteriorates the brain function due to decreased energy production (Solela et al. 2024). Chronic low oxygen is strongly linked with developing AD, as chronic hypoxia increases the expression of BACE1 and HIF-1α. This increased expression then leads to enhanced amyloidogenic processing along with Aβ accumulation in brain tissue, which is the central pathological mechanism involved in AD progression (Carey et al. 2024). Hypoperfusion has many effects on the whole body, including cardiovascular diseases, endothelial dysfunction, and blood-brain barrier permeability. Thus, this interplay between atherosclerosis, hypoperfusion, and T2D leads to the exacerbation of cognitive decline and cerebrovascular health (Guan et al. 2023).

Chronic high blood sugar levels significantly undermine the integrity of the BBB by markedly increasing its permeability and aiding the influx of noxious agents into the brain; this is therefore most catastrophic in AD as well as in T2D. The compromise is principally manifested by reduced LRP1 expression and increased RAGE-mediated signaling that antagonizes amyloid-β (Aβ) clearance. This leads to Aβ deposition and subsequent aggravation of neurodegeneration (Mayer and Fischer 2025). T2D also causes the formation of small infarcts and microbleeds culminating in cerebral small vessel diseases, which subsequently impair connectivity within the brain, contributing to cognitive decline (Uprety et al. 2021). White matter hyperintensities seen on MRI are associated with AD, underscoring vascular contribution to degenerative processes in the nerves (Lee and Funk 2023).

## Therapeutic frontiers: from bench to bedside

### Insulin sensitizers

The management goal in both T2D and AD is to address the insulin resistance. Numerous treatments targeted at increasing insulin sensitivity show surprising results in both diseases.

- **Intranasal Insulin**: This is the modern technique in which insulin is delivered directly to the brain via the nasal route. This not only enhances the metabolic function but also improves the memory function. The strategy is still promising, even though studies like the SNIFF trial have shown mixed results.
- **GLP-1 Agonists**: GLP-1 agonists include drugs like liraglutide and semaglutide. These drugs not only improve insulin sensitivity but also reduce the amyloid-beta (Aβ) accumulation and inflammation. Recently, many trials are being carried out, such as ELAD and EVOKE, to examine their impact on slowing AD progression.
- **Metformin and Thiazolidinediones (TZDs)**: These drugs improve the insulin sensitivity and lower oxidative stress, but the long-term effects of these drugs on cognitive decline are still being studied. These have been summarized in Table 5, given below.

**Table 5:** Emerging pharmacotherapies targeting shared metabolic and neurodegenerative pathways in T2D and AD

Therapy	Target Pathway	Stage	Key Trial/Study
Intranasal Insulin	Improves brain glucose metabolism, modulates Aβ clearance	Phase II	SNIFF Trial (Meglio 2024)
GLP-1 Agonists	Enhances insulin signaling, reduces Aβ/tau pathology	Phase III	ELAD Trial (Femminella et al. 2019)
MitoQ	Mitochondrial antioxidant reduces ROS damage	Preclinical	(Nguyen et al. 2020)
- **Emerging Insulin Mimetics and Sensitizers**: Apart from well-known insulin sensitizers such as metformin and GLP-1 receptor agonists, new insulin mimetics such as berberine have shown dual advantages in neurodegenerative as well as metabolic diseases. A plant-derived isoquinoline alkaloid, Berberine, activates AMP-activated protein kinase (AMPK), hence increasing glucose metabolism and improving insulin sensitivity. Moreover, preclinical research has shown neuroprotective effects by lowering tau hyperphosphorylation and amyloid-beta aggregation, therefore pointing to its potential for modifying the course of Alzheimer’s disease (Dan et al. 2024). These results highlight the therapeutic potential of metabolic agents that simultaneously address insulin resistance and neurodegeneration in cognitive decline associated with T2D.modifying the course of Alzheimer’s disease (Dan et al. 2024). These results highlight the therapeutic potential of metabolic agents that simultaneously address insulin resistance and neurodegeneration in cognitive decline associated with T2D.

### Anti-Inflammatory strategies

Due to the major impact of inflammation on T2D and AD, much work has been done on anti-inflammatory techniques. The therapies that show promising results in reducing neuroinflammation and cognitive decline include SGLT2 inhibitors, TNF-α inhibitors, and natural anti-inflammatory substances.

- **TNF-α Inhibitors**: The TNF-α inhibitor etanercept has shown surprisingly major cognitive improvements in small-scale studies done on AD patients. This indicates the possibility of its wider therapeutic uses in neurodegenerative diseases (Paul et al. 2024).
- **SGLT2 Inhibitors**: Empagliflozin is mostly used to regulate blood sugar levels in T2D patients, and has also shown anti-inflammatory properties by lowering pro-inflammatory mediators in activated microglia. While in the case of AD, this might slow down the neurodegenerative process, and currently, many studies are being done to confirm this additional benefit (Heimke et al. 2022). This class of drug controls inflammation via ERK1/2 and NFκB pathways and suggests a dual function in the treatment of neuroinflammation and diabetes.
- **Repurposing Conventional Anti-Inflammatory Drugs**: In preclinical models, nonsteroidal anti-inflammatory drugs (NSAIDs) such as ibuprofen have proven neuroprotective effects by lowering proinflammatory cytokines, suppressing microglial activation, and decreasing amyloid plaque burden—partly through suppression of glutamate-induced excitotoxicity (Yan et al. 2003). Another NSAID, naproxen, similarly lowers inflammation, though its effectiveness in AD has not been well described. Colchicine has been found to block NLRP3 inflammasome activation, therefore lowering IL1β production and neuroinflammation (Ajmone-Cat et al., 2010). In preclinical models of AD, the tetracycline antibiotic minocycline inhibits microglial activation and lessens oxidative stress, therefore indicating a possible benefit in modulating neuroinflammatory cascades (Ajmone-Cat et al. 2010). Another likely candidate is masitinib, a selective tyrosine kinase inhibitor presently in clinical trials that controls neuroimmune signaling and suppresses mast cell–mediated inflammation, therefore providing a novel method to lessen central immune dysregulation in AD (Ajmone-Cat et al. 2010). These results point out the potential of repurposing well-defined anti-inflammatory medications for dual use in metabolic and neurodegenerative diseases.
- **Natural Anti-Inflammatory Compounds**: Many natural compounds high in flavonoids and polyphenols, such as curcumin and resveratrol, have anti-inflammatory and antioxidant qualities. But, their bioavailability is the major problem of their use in therapeutic medicine (Paul et al. 2024). Different studies done on these natural compounds have shown their potential in reducing oxidative stress and inflammation in the brain tissue, proving them promising in the treatment of AD.

### Mitochondrial therapies

In both AD and T2D, dealing with mitochondrial dysfunction is very important for preserving brain health and function. In managing mitochondrial dysfunction, two mitochondria-targeted antioxidants, MitoQ and SS-31, have shown promising results in lowering oxidative damage and enhancing cellular energy (Paul et al, 2021). Secondly, regular exercise also promotes mitochondrial health. Regular exercise through various mechanisms activates irisin and brain-derived neurotrophic factor (BDNF), hence enhancing mitochondrial biogenesis. Additionally, the use of two NAD + precursors such as nicotinamide mononucleotide (NMN) and nicotinamide riboside (NR) have also shown neuroprotective effects by improving mitochondrial function (Atlante et al. 2022) These therapies not only reduce the mitochondrial dysfunction seen in both conditions, but also improve the cognitive decline and its progression (Kalani et al. 2023).

Other mitochondrial-targeted antioxidant treatments have shown encouraging results in both T2D and AD, in addition to MitoQ and SS31. Alphalipoic acid (ALA) operates by scavenging reactive oxygen species (ROS), chelating metals, and reestablishing natural antioxidants like glutathione, therefore increasing whole cellular antioxidant capacity. Moreover, it enhances insulin sensitivity, which is especially helpful in T2D-linked cognitive impairment (Superti and Russo 2024). Melatonin also has strong antioxidant effects, stabilizes mitochondrial membranes, and has been found to lower tau phosphorylation, a major driving force behind Alzheimer’s disease (Morén et al. 2022). These drugs have great therapeutic promise via dual effect on oxidative stress and metabolic control.Other mitochondrial targeted antioxidant treatments have shown encouraging results in both T2D and AD in addition to MitoQ and SS31. Alphalipoic acid (ALA) operates by scavenging reactive oxygen species (ROS), chelating metals, and reestablishing natural antioxidants like glutathione, therefore increasing whole cellular antioxidant capacity. Moreover, it enhances insulin sensitivity, which is especially helpful in T2D-linked cognitive impairment (Superti and Russo 2024). Melatonin also has strong antioxidant effects, stabilizes mitochondrial membranes, and has been found to lower tau phosphorylation, a major driving force behind Alzheimer’s disease (Morén et al. 2022). These drugs have great therapeutic promise via dual effect on oxidative stress and metabolic control.

### Precision medicine & future directions

Precision medicine, in which treatment is given according to each patient’s unique genetic and metabolic profile, offers a revolutionary approach in managing neurodegenerative disorders, including AD and T2D. Genetic profiling can help in doing targeted interventions by identifying risk-related genes like the APOE ε4 variant. This maximizes therapeutic success in the management of AD (Chang et al. 2022). Due to the multifactorial nature of AD and T2D, a combination of metabolic, anti-inflammatory, and mitochondrial support medications may also be beneficial in treating both conditions (Rossi et al. 2023). Longitudinal studies are also essential for monitoring the course of the disease, which will assist in identifying the best times for interventions and improve treatment plans (Schork and Elman 2023). This strategy highlights how precision medicine has the potential to completely transform the management of neurodegenerative illnesses, as summarized in Table 6.

**Table 6:** Integrated lifestyle and Pharmacological interventions for dual management of T2D and AD

Recommendation	Mechanistic Basis	Evidence Level
Mediterranean Diet	Reduces AGEs, enhances insulin sensitivity, lowers inflammation	Meta-analysis (Grade A) (Zooravar et al. 2025)
Aerobic Exercise	↑ Mitochondrial biogenesis, (PGC-1α) enhances neuroplasticity	RCTs (Grade B) (Wu et al. 2024)
SGLT2 Inhibitors	↓ Neuroinflammation, protects the BBB, enhances ketone metabolism	Preclinical/Phase II (Dabour et al. 2024)

## Conclusion

The interplay between T2D and AD reveals a complex overlap of shared metabolic and inherently degenerative factors and mechanisms, such as resistance to insulin, inflammation, oxidative injury, mitochondrial and vascular dysfunction. Together, these processes are responsible for the amyloid-beta accumulation, tau hyperphosphorylation, and destruction of synapses, leading to progressive loss of cognition. To tackle the multifaceted problem of T2D and AD, an integrated strategy with behavioral changes, drug treatment, and targeted approaches is needed. Effective caring for people at risk requires accurate early diagnosis and timely intervention to preserve metabolic and cognitive resilience in affected individuals.The interplay between T2D and AD reveals a complex overlap of shared metabolic and inherently degenerative factors and mechanisms such as, resistance to insulin, inflammation, oxidative injury, mitochondrial and vascular dysfunction. Together these processes are responsible for the amyloid-beta accumulation, tau hyperphosphorylation and destruction of synapses leading to progressive loss in cognition. To tackle the multifaceted problem of T2D and AD, an integrated strategy with behavioral changes, drug treatment, and targeted approaches is needed. Effective caring for people at risk requires accurate early diagnosis and timely intervention to preserve metabolic and cognitive resilience in affected individuals.

Emerging studies suggest that GLP-1 agonists, intranasal insulin, and mitochondrial antioxidants have dual promise for metabolic and cognitive health. But their efficacy is dependent on early intervention, making them need plasma p-tau/Aβ42 ratio biomarkers for early diagnosis. Also, making lifestyle changes like eating a Mediterranean diet and getting regular aerobic exercise can improve insulin sensitivity and lower oxidative stress. All of these support metabolic and cognitive health and also encourage neuroplasticity.

In summary, the effective management of T2D and AD need a comprehensive and multidisciplinary restructuring of care systems at the organizational level. By focusing on integrated systems of intervention and utilizing new biomarker and personalized medicine techniques, the public health challenge posed by these two epidemics can be lessened while simultaneously encouraging a future with ideal metabolic and cognitive outcomes.

## Data Availability

No datasets were generated or analysed during the current study.
